# Changes in the Structure of Strawberry Leaf Surface Bacterial and Fungal Communities by Plant Biostimulants

**DOI:** 10.3390/microorganisms13112461

**Published:** 2025-10-28

**Authors:** Ji Yoon Lee, Hyeran Shin, Juhyun Yu, Hyun Gi Kong

**Affiliations:** 1 Department of Plant Medicine, College of Agriculture, Life & Environment Sciences, Chungbuk National University, Seowon-gu, Cheongju 28644, Republic of Korea; 2 SugarArt Research Institute, Chungju 28181, Republic of Koreajhyu5102@hanmail.net (J.Y.)

**Keywords:** plant biostimulant, phyllosphere, microbiome, strawberry

## Abstract

The application of plant biostimulants to enhance fruit quality is increasing, yet their impact on the phyllosphere microbiome remains understudied. This study investigated the effects of a sugar-based biostimulant on the bacterial and fungal communities on strawberry leaf surfaces using Illumina MiSeq sequencing. The sweetener treatment significantly decreased bacterial alpha diversity (Shannon and Simpson indices). Compositional analysis revealed a lower relative abundance of the phylum *Pseudomonadota*, whereas the fungal phylum *Ascomycota* increased and *Basidiomycota* decreased. At the family level, *Sphingobacteriaceae*, *Bacillaceae*, and *Micrococcaceae* were significantly enriched in the treated group. Furthermore, we isolated bacterial strains, including *Sphingomonas zeae* St1 and *Frigoribacterium faeni* TSAY2, which increased in abundance post-treatment and demonstrated enhanced growth using the sweetener as a sole nutrient source. These findings suggest that sugar-based biostimulants directly reshape the composition and functional potential of the phyllosphere microbiome, which may, in turn, influence nutrient uptake, plant growth, and immunity.

## 1. Introduction

Fruits provide a major contribution to the human diet, supplying essential vitamins, carbohydrates, and water [1,2]. Among fruit crops, strawberries represent one of the most extensively cultivated berries worldwide. Modern cultivars are now grown in diverse climates, from temperate to tropical regions [3]. The garden strawberry (*Fragaria* × *ananassa*) is a relatively recent crop, having originated roughly three centuries ago through hybridization between the North American species Fragaria virginiana and the South American species Fragaria chiloensis [4]. Subsequent breeding programs in the 19th century enhanced desirable traits such as fruit size, firmness, and yield, which fueled the rapid expansion of strawberry cultivation across Europe and the United States [5]. As of 2023, global strawberry production exceeded 10 million tons, with Asia accounting for over half of the total output, followed by the Americas (22.8%) and Europe (17%) [6].

Consumer acceptance of strawberries is primarily determined by attributes related to fruit quality, including size, shape, firmness, sweetness, and overall flavor [7,8]. In particular, sweetness and texture exhibit strong correlations with consumer preference [9]. The perceived flavor of strawberries is influenced mainly by soluble sugars, notably sucrose and glucose, together with organic acids [10,11]. These compounds, along with other metabolites, are subject to dynamic regulation during the ripening process [12]. Previous metabolomic analyses have confirmed that sucrose, glucose, and fructose are the dominant soluble sugars in strawberry fruits [13,14].

Because conventional breeding has limitations in terms of improving flavor traits, external interventions have been investigated as complementary approaches. Plant biostimulants, including natural sweeteners such as stevia extracts, amino acid formulations, and other biological elicitors, can modulate metabolic processes to enhance flavor. Similarly, exogenous application of sugars has been reported to improve both stress tolerance and physiological responses in plants by influencing metabolism and gene expression. For instance, trehalose treatment has been shown to promote growth and photosynthetic efficiency while strengthening antioxidant defenses [15]. Chitosan oligosaccharides have been demonstrated to reduce lipid peroxidation in cucumber (*Cucumis sativus* L.) and to stimulate the expression of antioxidant enzymes and heat shock proteins [16]. In strawberries, exogenous fructose application has been associated with increased fruit weight, higher soluble solids, and elevated sugar content, accompanied by transcriptomic and metabolomic evidence of enhanced expression of sugar metabolism genes such as FaINV and FaSUS [17].

Since metabolite accumulation is also influenced by plant-associated microbes, treatments that modify plant metabolism may concurrently alter microbial communities. Recent studies suggest that these chemical traits are not only genetically and physiologically regulated but are also influenced by interactions with plant-associated microbial communities [11,18]. Microorganisms inhabiting plant surfaces and tissues play critical roles in plant phenotype, metabolism, and stress resilience [19,20,21]. In strawberries, microbial community composition varies across developmental stages and plant organs, with *sphingomonas* consistently reported as a dominant bacterial group in the phyllosphere [18]. Despite this, the effects of pre-harvest sugar treatments on strawberry-associated microbiota remain poorly understood. Given the complexity of phyllosphere communities and their potential to influence plant metabolism, investigating these interactions is increasingly important.

In this study, we evaluated how exogenous sugar treatments influence strawberry fruit sweetness throughout development and examined their effects on the composition of bacterial and fungal communities colonizing leaf surfaces. Identifying the interplay between external treatments and the phyllosphere microbiome provides insights into sustainable strategies for enhancing fruit flavor and quality, with potential benefits for postharvest shelf life and consumer acceptance.

## 2. Materials and Methods

### 2.1. Strawberry Cultivation and Sample Collection

The experiment was conducted in a commercial strawberry greenhouse located at coordinates 35.2871439, 127.9286919. Strawberry plants (*Fragaria × ananassa* Duch., cv. Seolhyang) were cultivated in elevated beds filled with peat moss substrate. Seedlings were transplanted in October at 15 cm intervals. Plants were grown for seven months before being used in the experiment. Each cultivation bed represented one replicate, with five biological replicates per treatment, and 15 plants were grown in each bed. The nutrient solution supplied to the plants was adjusted to an electrical conductivity (EC) of 1.0 dS·m^−1^ and a pH of 6.0. During the experimental period, the mean temperature, relative humidity, and daily sunshine duration in the greenhouse were 17.8 ± 2.5 °C, 67.2 ± 10.7%, and 8.2 ± 4.2 h, respectively.

The sugar-enhancing agent (SugarPump, SugarArt Co., Cheongju-si, Republic of Korea) consisted of 30% high fructose, 3% adhesive agents, 2% phosphates, 1% sulfates, 0.1% boric acid, and 0.005% sodium molybdate. The solution was diluted 100-fold and applied to the plants at a rate of 50 mL per plant. Treatments were conducted three times at three-day intervals. Strawberry fruits and leaves were harvested three days after the third treatment and used for the study.

**Figure 1:**
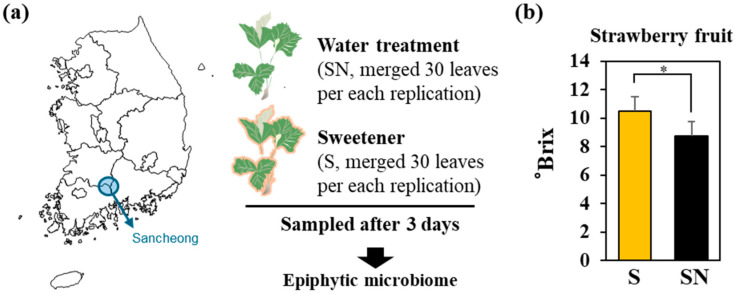
Strawberry sampling and measurement of phyllosphere microbiome composition. (**a**) Overview of the sampling site and description of leaf sample collection for microbiome analysis. (**b**) Comparison of soluble sugar content in strawberry flesh between sugar-enhancer–treated (S) and water treated (SN) groups. Statistical significance was determined using a Student’s *t*-test (* = *p* < 0.05).

Mature fruits were harvested to determine soluble solid content (°Brix). Fruit juice from 30 berries per cultivation bed was pooled, and measurements were performed across five replicates. A schematic overview of the experimental design is shown in Figure 1a.

For phyllosphere microbiome analysis, leaf samples were collected three days after the third application of the biostimulant (S). Water-treated plants (*n* = 5) served as the control (SN). Only visually healthy leaves without any disease symptoms were used, and newly emerged leaves after the treatment were excluded. Thirty leaves were collected per bed and washed in 10 mL of phosphate-buffered saline (PBS) to extract surface microorganisms. The pooled suspensions were then subjected to phyllosphere microbial community analysis. Each treatment consisted of five biological replicates based on cultivation beds.

### 2.2. DNA Extraction, Sequencing, and Data Processing

DNA was extracted from leaf suspensions using the FastDNA™ Spin Soil Kit (MP Biomedicals, Eschwege, Germany). Concentration and purity were assessed with a NanoDrop NC2000 spectrophotometer (Thermo Fisher Scientific, Seoul, Republic of Korea).

For amplicon sequencing, 2 ng of DNA was used in PCR reactions containing 5× reaction buffer, 1 mM dNTPs, 500 nM forward and reverse primers, and DNA polymerase (Agilent Technologies, Santa Clara, CA, USA). The bacterial 16S rRNA V3–V4 region and fungal ITS region was amplified. Primers used were: V3-F (5′-TCGTCGGCAGCGTCAGGATGTAAGACAGCCTAGGGGNGGCWGCAG-3′) and V4-R (5′-GTCTCGTGGGCTCGGAGATGTGTATAAGAGACAGGACTACHVGGGTATCTAATCC-3′) for bacteria; ITS3-F (5′-GCATCGATGAAGAACGCAGC-3′), ITS4-R (5′-TCCTCCGCTTATTGATATGC-3′) for fungi. PCR products were purified with AMPure beads, and fragment size and quality were confirmed using a TapeStation D1000 system (Agilent Technologies, USA). Sequencing was performed on an Illumina MiSeq™ platform (Illumina, San Diego, CA, USA).

Raw sequences were processed in QIIME 2 (v2025.4) [22]. Demultiplexing was conducted using the demux plugin, primers were trimmed with Cutadapt (v3.2) [23], and paired-end reads were merged, quality-filtered, and chimera-removed using DADA2 [24]. Taxonomic assignment of amplicon sequence variants (ASVs) was performed against the SILVA v138.2 database [25]. All samples were rarefied to 70,000 reads before downstream analyses.

Alpha diversity indices, including Shannon, Simpson, observed features, and ACE, were calculated. The number of observed ASVs was also recorded. Multiple sequence alignment was performed with MAFFT (v7.475), and phylogenetic trees were generated using FastTreeMP (v2.1.11). To ensure adequate sequencing depth, rarefaction curves were examined prior to diversity analyses, and read counts were normalized accordingly. Beta diversity was estimated using Bray–Curtis dissimilarity. Non-metric Multidimensional Scaling (NMDS) was applied to visualize between-sample variation. Heatmaps of bacterial relative abundances were constructed in R (v4.5.1). Ecological functions were assigned using FAPROTAX (v1.2.2) [26]. The microbiome data were registered in the NCBI database; the related Submission ID is SUB15708182 and the BioProject ID is PRJNA1345213.

### 2.3. Isolation of Treatment-Specific Bacteria from Strawberry Leaves

Changes in culturable microorganisms on strawberry leaves following the application of the sugar-enhancing agent were investigated. The same leaves used for microbiome analysis were processed for microbial cultivation. Leaf discs (2 cm in diameter) were excised using a sterile cork borer and placed in 50 mL tubes containing 5 mL of sterile distilled water. The samples were vortexed to detach microorganisms from the leaf surface. The resulting leaf suspensions were serially diluted up to 10^−3^, and 100 μL aliquots of each dilution were spread onto tryptic soy agar (TSA; BD Difco^TM^, Franklin Lakes, NJ, USA) for bacterial growth and potato dextrose agar (PDA; BD Difco^TM^, NJ, USA) for fungal growth. Plates were incubated at 25 °C for 3 days. Leaves treated with water were used as controls, consistent with the microbiome analysis. Five leaves per treatment were used, and colony-forming units (CFUs) were recorded for each leaf to quantify culturable microbial populations. Comparative analysis was performed on dilutions that produced fewer than 100 colonies.

Colonies that appeared exclusively in treated plants (based on morphology and pigmentation) were selected as treatment-specific isolates. These isolates were subcultured twice on TSA, suspended in 20% glycerol, and stored at −80 °C. Genomic DNA was extracted with the GeneAll^®^ Exgene™ Cell SV kit (GeneAll Biotechnology, Seoul, Republic of Korea) for PCR assays.

### 2.4. Identification of Bacterial Isolates

PCR amplification was performed in 20 μL reactions containing 2 μL DNA (50 ng/μL), 2 μL 10× buffer, 0.4 μL 10 mM dNTPs, 1 μL of each primer (10 μM), 0.1 μL Taq DNA polymerase (BioFact, Daejeon, Republic of Korea), and 13.5 μL DNase/RNase-free distilled water (Invitrogen). The 16S rRNA gene was amplified using primers 27F (5′-AGAGTTTGATCCTGGCTCAG-3′) and 1492R (5′-TACGGYTACCTTGTTACGACTT-3′). PCR conditions: initial denaturation at 96 °C for 4 min; 30 cycles of 94 °C for 30 s, 57 °C for 30 s, and 72 °C for 1 min; final extension at 72 °C for 10 min. Products were confirmed on 1.5% agarose gels (0.5× TBE buffer) and sequenced bidirectionally (BioFact, Daejeon, Republic of Korea). Sequences were aligned against the NCBI database using BLAST. Phylogenetic trees were built with MEGA 11 (v11.0.13) using the Neighbor-Joining method and 1000 bootstrap replicates. *Agrobacterium arsenijevicii* KFB 330 served as the outgroup.

### 2.5. Growth Assay of Isolates with Sugar-Enhancing Biostimulant

Three treatment-specific bacterial isolates were tested for growth using a biostimulant as a single nutrient. As a control, growth was observed by culturing in PBS. Isolates were cultured on TSA at 25 °C for 24 h. The biostimulant was diluted 1:250 in PBS bufffer (final concentration: 4 μL/mL) and filtered through a 0.2 μm syringe filter (Dismic-25, ADVANTEC, Tokyo, Japan). Bacterial suspensions were added to the filtered solution, and the cultures were prepared using a UV/Vis spectrophotometer (Nabi, Seoul, Republic of Korea) and Macro Vis Cuvettes (1000 μL, Eppendorf, Germany) to an OD_600_ of 0.1. Each culture was inoculated into a 14 mL tube (SPL Life Sciences, Seoul, Republic of Korea) by adding 1 mL of the bacterial suspension to 9 mL of the filtered solution, resulting in a 10-fold dilution. OD_600_ was measured at 0 h, followed by incubation at 28 °C, 150 rpm for 24 h, and re-measured. The bacterial suspension process was performed in a sterile atmosphere. The OD_600_ values for each isolate in each treatment were compared and analyzed. For growth analysis, three experimental repetitions were performed.

### 2.6. Statistical Analysis

All statistical analyses were performed using R software (version 4.3.1). For microbiome analysis, each treatment included five biological replicates (*n* = 5). For growth assays of isolates, three biological replicates were performed. Prior to statistical analyses, the data were examined for normality using the Shapiro–Wilk test and for homogeneity of variances using Levene’s test. When the data satisfied both assumptions, differences among treatments were analyzed using one-way analysis of variance (ANOVA) followed by Tukey’s honestly significant difference (HSD) test for multiple comparisons. The significance level was set at *p* < 0.05. In cases where the assumptions were not met, a non-parametric Kruskal–Wallis test followed by Dunn’s post hoc test was applied. Because the number of comparisons was limited within each analysis, no additional correction (e.g., Bonferroni or false discovery rate adjustment) was applied. Statistical differences in community composition among groups were evaluated using PERMANOVA with 999 permutations by R program (v4.5.1).

## 3. Results

### 3.1. Effects of the Plant Biostimulant on Fruit Sugar Content and Relative Abundance of Microbial Taxa

At a commercial strawberry farm in Sancheon, Gyeonggi Province, a plant-based sweetness enhancer was applied three times at one-week intervals. One week after the final application, fruit sugar content and leaf-associated microbiome composition were assessed (Figure 1a). The soluble solids content of strawberry juice, measured as °Brix, significantly increased in treated plants (S) compared to the water treated control (SN). Control plants averaged 8 °Brix, whereas treated fruits exceeded 10 °Brix (Figure 1b).

Leaves from five replications per treatment were analyzed for bacterial and fungal community composition. The initial reads generated in the bacterial microbiome analysis ranged from 69,569 to 94,816, and quality filtering and non-chimeric removal processes ultimately secured over 82% of the reads. The fungal microbiome analysis also generated reads ranging from 82,648 to 118,995, and after analysis, over 67% of the reads were used in the final analysis (Supplementary Table S1).

**Figure 2:**
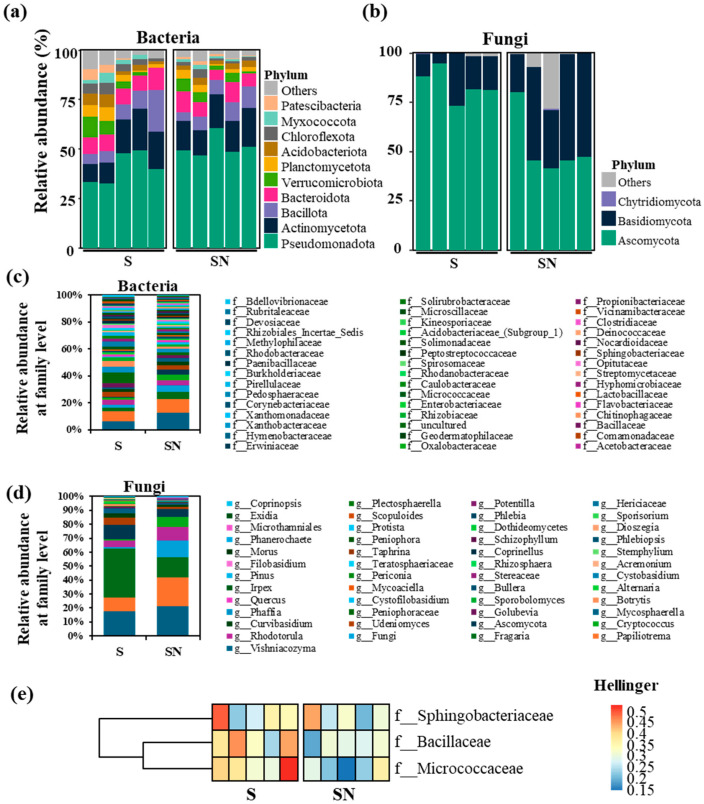
Relative abundance of strawberry phyllosphere microbial communities. (**a**,**b**) Bacterial and fungal communities at the phylum level. (**c**,**d**) Bacterial communities at the family level. Relative abundance values are expressed as percentages. (**e**) The abundance of sphingobacteriaceae, bacillaceae, and micrococcaceae, which showed differences at the family level, was compared between samples using the Hellinger index. The sugar content enhancer treatment is indicated as S, and the water treatment as a control is indicated as SN.

At the phylum level, bacterial communities in treated plants showed higher relative abundances of Bacillota, Planctomycetota, and Patescibacteria (Figure 2a). Fungal communities displayed an increase in Ascomycota and a reduction in Basidiomycota (Figure 2b). At the family level, bacterial taxa such as Pedosphaeraceae, Chitinophagaceae, and Rhizobiaceae increased, whereas Pseudomonadaceae and Erwiniaceae decreased (Figure 2c). Among fungi, genera associated with Fragaria and Udeniomyces increased, while Cryptococcus decreased (Figure 2d). Further analysis of less abundant bacterial families revealed increases in Sphingobacteriaceae, Bacillaceae, and Micrococcaceae in the treatment group (Figure 2e).

### 3.2. Microbial Diversity in Strawberry Leaves After Treatment

**Figure 3:**
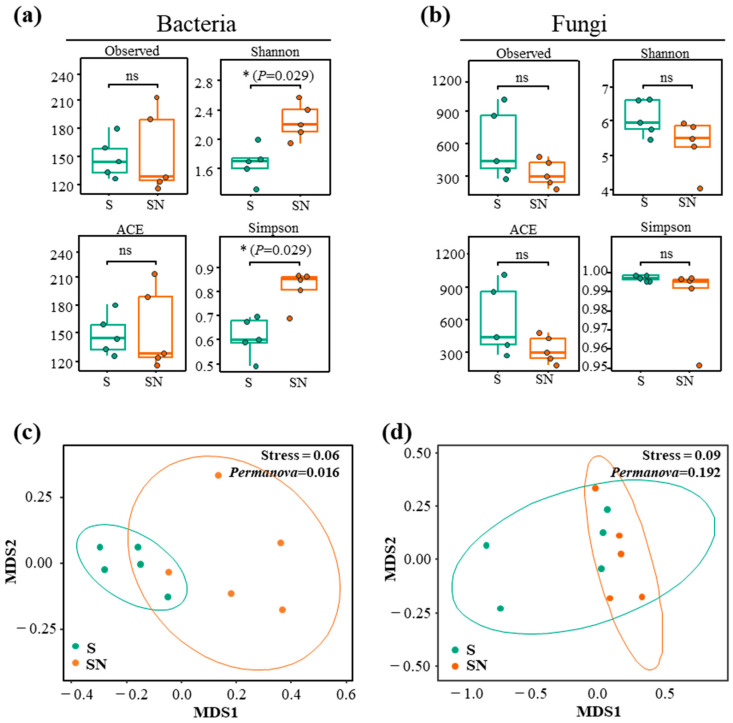
Diversity analyses of strawberry leaf bacterial and fungal communities. (**a**) Alpha diversity indices (observed features, Shannon, ACE, Simpson) for bacterial communities. Statistical significance was determined using a Student’s *t*-test (ns = not significant, * = *p* < 0.05). (**b**) Alpha diversity indices for fungal communities. (**c**) Non-metric multidimensional scaling (NMDS) of Bray–Curtis dissimilarities for bacterial communities. (**d**) NMDS of Bray–Curtis dissimilarities for fungal communities. Significant pairwise differences between treatment (S) and control (SN) are indicated (*p* < 0.05). Red = water treated (SN); green = treated (S).

Alpha diversity metrics were used to evaluate community diversity and evenness. In bacteria, the number of observed features and the ACE index did not differ significantly between groups; however, both Shannon and Simpson indices significantly decreased in treated plants (*p* = 0.029; Figure 3a).

For fungi, observed features and ACE values increased in the treatment group, but differences were not statistically significant. Likewise, Shannon and Simpson indices did not differ between groups (Figure 3b). Beta diversity analysis by NMDS showed that bacterial communities differed significantly between treatments (PERMANOVA *p* = 0.016), with treated samples clustering more tightly (Figure 3c). Fungal communities, however, showed no significant separation between groups (PERMANOVA *p* = 0.192; Figure 3d).

### 3.3. Random Forest Analysis of Key ASVs

**Figure 4:**
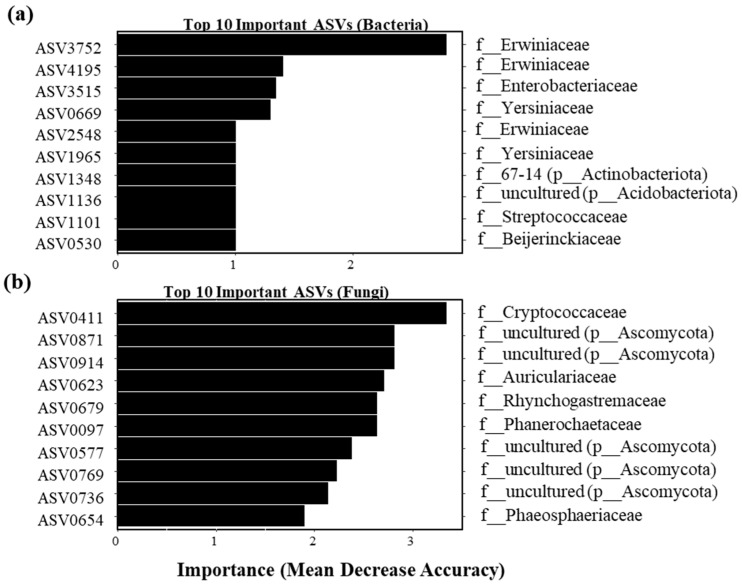
Random forest classification of phyllosphere microbial communities. Importance scores of the top 10 discriminatory ASVs are shown. (**a**) Bacterial ASVs distinguishing treated (S) and water treated (SN) plants. (**b**) Fungal ASVs distinguishing treated and water treated plants.

Random forest classification identified the top 10 amplicon sequence variants (ASVs) that discriminated between treatments (Figure 4). For bacteria, ASV3752 (f_Erwiniaceae) had the highest importance score (Mean Decrease Accuracy; Figure 4a). For fungi, ASV0411 (f__Cryptococcaceae) was the most significant discriminatory ASV. Consistent with community-level analysis, erwiniaceae abundance decreased in treated plants (Figure 2c), while p__Ascomycota increased (Figure 2b).

### 3.4. Functional Features of the Phyllosphere Microbiota

**Figure 5:**
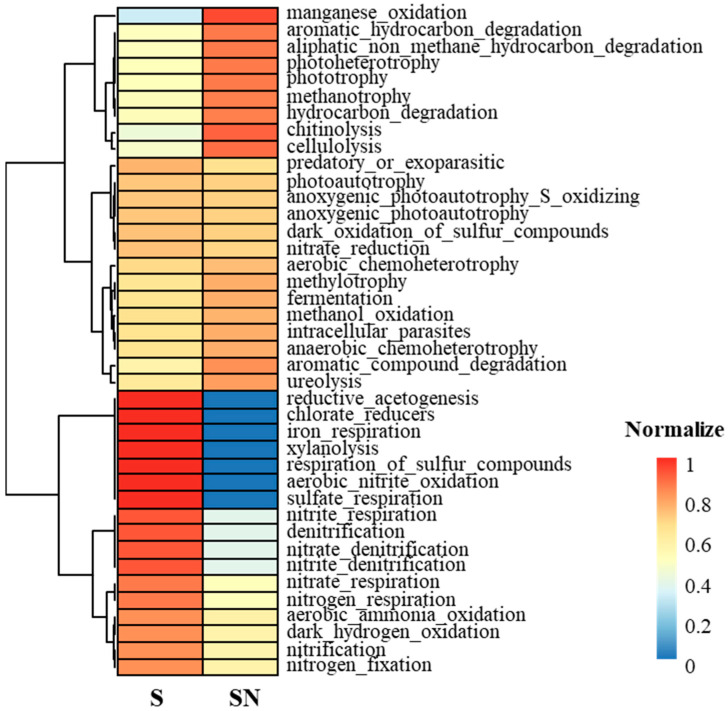
Predicted functional features of the phyllosphere microbiota using FAPROTAX. Functional categories are shown for water treated (SN) and treated (S) samples. Colors represent relative abundance (red = higher abundance; blue = lower abundance). Samples were normalized and clustered to display group-level differences.

FAPROTAX functional prediction identified 40 categories, including metabolic and fermentative functions (Figure 5). In treated plants, functions associated with nitrate, nitrogen, and sulfur respiration, as well as xylan degradation, significantly increased. In contrast, hydrocarbon degradation, phototrophy, chitin degradation, and cellulose degradation decreased. Also, redundancy analysis (RDA) revealed significant correlations between fruit Brix values and both fungal and bacterial community compositions. Among the fungal taxa, ascomycota (ASV0577) showed a strong positive association with Brix. Within the bacterial community, members of the families beijerinckiaceae (ASV2112, ASV1883, ASV0304) and sphingomonadaceae (ASV1491) were also significantly correlated with fruit sweetness (Supplementary Figure S1). The beijerinckiaceae and sphingomonadaceae were positively associated with higher Brix values, suggesting a potential role in sugar metabolism.

### 3.5. Cultivation-Based Analysis of Leaf Microbes

**Figure 6:**
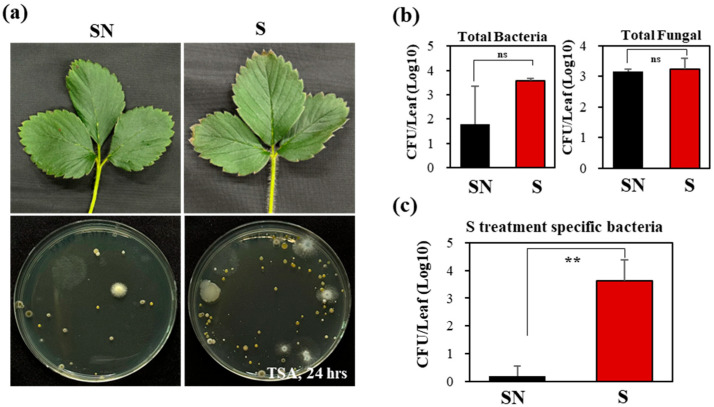
Culture-dependent analysis of strawberry leaf microorganisms following sugar-enhancer treatment. (**a**) Leaf morphology, microbial isolation, and colony growth on TSA medium after 24 h at 25 °C. (**b**) Total bacterial and fungal colony-forming units (CFU) per leaf. (**c**) Bacterial CFU counts of selected strains. Statistical analysis: *p* < 0.01 (**), not significant (ns, *p* > 0.05).

Culturable bacterial and fungal populations were also assessed. Plant biostimulant treatment did not cause visible physiological changes in leaves (Figure 6a). Total CFUs per leaf were higher in treated plants (3.5 × 10^3^ CFU/leaf) compared with controls (1.8 × 10^2^ CFU/leaf), although the difference was not statistically significant (Figure 6b). Similarly, fungal populations were comparable between groups (3.2 × 10^3^ vs. 3.1 × 10^3^ CFU/leaf). Notably, a subset of bacterial strains was significantly enriched in treated plants (Figure 6c).

**Figure 7:**
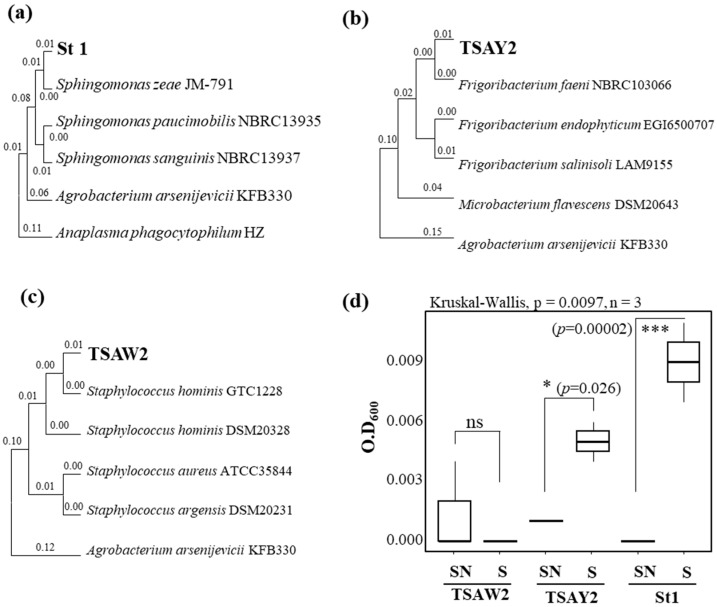
Growth responses of isolated bacterial strains to sugar enhancers. (**a**–**c**) Phylogenetic tree of bacterial isolates based on nearly full-length 16S rRNA sequences, constructed using the neighbor-joining method in MEGA X. Accession numbers of isolated strains are shown in bold. (**d**) In vitro growth analysis of isolates with or without sugar enhancer supplementation under nutrient-free conditions. Significant differences between treatments are indicated (*** *p* < 0.0001, * *p* < 0.05, ns = not significant).

Colony characteristics of the three strains that specifically appeared in the sweetener were as follows. For the St1 strain, the colonies were generally bright yellow and smooth, measuring 2 + 1 mm in diameter after 24 h of incubation in TSA. The colonies were generally convex, with raised edges that may appear smooth overall. The colonies of the TSAY2 isolate were generally yellowish, almost white, and small, measuring 1 mm in diameter. TSAW2 was a circular, cream-colored to white colony, measuring approximately 1 mm in diameter. The colony center was opaque, and the border was transparent. Three isolates from the treatment group were identified through 16S rRNA sequencing as *Sphingomonas zeae* St1, *Frigoribacterium faeni* TSAY2, and *Staphylococcus hominis* TSAW2 (Figure 7a–c). Growth assays under nutrient-free conditions showed that *S. zeae* St1 exhibited the strongest growth response when sweeteners were present (*p* = 0.00002). *F. faeni* TSAY2 also showed a significant increase (*p* = 0.026), whereas *S. hominis* TSAW2 did not (Figure 7d). All representative 16S rRNA gene sequences obtained in this study have been submitted to GenBank (SUB15713084) and have been accessioned under the accession numbers *Sphingomonas zeae* St1 (PX460783), *Frigoribacterium faeni* TSAY2 (PX460784), and *Staphylococcus hominis* TSAW2 (PX460785).

## 4. Discussion

The present study demonstrates that foliar application of a plant-based sweetness enhancer can simultaneously improve fruit quality and alter the phyllosphere microbial community in strawberries. The increase in fruit °Brix under field conditions indicates that the treatment effectively enhanced carbohydrate accumulation, consistent with previous findings that exogenous sugars or sugar-rich biostimulants can promote source–sink transport and stimulate sucrose metabolism in fruits [15,17].

Beyond sweetness improvement, the significant shifts observed in the leaf microbiome suggest that the biostimulant influenced not only plant physiology but also the ecological environment of the phyllosphere. Changes in microbial composition, particularly within bacterial communities, indicate that nutrient availability and carbon source input from the foliar treatment selectively favored certain taxa.

The increase in specific bacterial families—sphingobacteriaceae, bacillaceae, and micrococcaceae—suggests that the altered nutrient environment promoted selective microbial growth. Members of these families are known for producing plant growth regulators, improving stress tolerance, and enhancing nutrient assimilation [27,28,29,30].

Thus, their proliferation may indirectly support plant performance following sweetness enhancer application. Conversely, the decline in Erwiniaceae, a family that includes potential phytopathogens such as *Erwinia* spp., suggests a possible protective effect of the treatment, either through competitive exclusion or through microbially mediated antagonism.

Microbial diversity metrics provide further insight into community restructuring. The observed reduction in bacterial evenness, reflected by lower Shannon and Simpson indices, indicates dominance by a few functionally active taxa. Beta diversity analyses confirmed distinct microbial assemblages between treated and control groups, suggesting a consistent selection pressure imposed by the enhancer. In contrast, fungal communities appeared more stable, showing minor shifts in the relative abundance of Ascomycota and Basidiomycota but without significant changes in overall diversity. This differential response implies that bacteria, which more rapidly utilize available carbon substrates, were more sensitive to the treatment than fungi.

Functional prediction and RDA analyses further support the interpretation that the treatment affected microbial metabolism and its relationship with fruit quality. Increased representation of nitrogen- and sulfur-related metabolic pathways among bacteria suggests enhanced nutrient turnover in the phyllosphere, potentially facilitating improved nutrient assimilation and photosynthetic performance in the host.

The significant correlations identified between fruit Brix and the abundance of Beijerinckiaceae and Sphingomonadaceae highlight the possibility that these bacterial taxa contribute to carbohydrate metabolism or influence plant sugar allocation through signaling interactions. Previous studies have reported that *Sphingomonas* species can modulate plant metabolic activity and improve fruit quality through biofilm formation and the production of secondary metabolites [31], supporting our findings.

Isolation of *Sphingomonas zeae* and *Frigoribacterium faeni* under sweetener-amended conditions provides culture-based evidence for the selective enrichment observed in the sequencing data. These bacteria may utilize components of the biostimulant as carbon sources or thrive in the modified microenvironment created by enhanced plant exudation. Their proliferation could stabilize the phyllosphere community and contribute to plant physiological benefits through nutrient exchange or stress mitigation. Together, these findings indicate that foliar sweetness enhancers have broader ecological consequences beyond fruit flavor enhancement, influencing microbial dynamics that may, in turn, reinforce plant performance.

Overall, our results support the idea that biostimulant-induced improvements in fruit quality are not solely physiological responses of the plant but involve integrated interactions between plant metabolism and the phyllosphere microbiome. Understanding these interactions may enable more targeted use of biostimulants to optimize both fruit quality and plant–microbe symbioses.

## 5. Conclusions

This study underscores the dual impact of foliar sweetness enhancers: direct improvement of fruit quality and indirect modification of leaf microbial communities. While some changes may be beneficial (e.g., pathogen suppression, plant growth promotion), the dominance of certain taxa could also introduce risks if opportunistic pathogens proliferate. Future work should investigate (1) the metabolic interactions between enhancers and microbial communities, (2) functional gene contributions of enriched strains, and (3) long-term impacts of repeated treatments on microbiome stability and plant health.

## Data Availability

The microbiome data were registered in the NCBI database; the related Submission ID is SUB15708182 and the BioProject ID is PRJNA1345213.

## Supplementary Materials

The following supporting information can be downloaded at: https://www.mdpi.com/article/10.3390/microorganisms13112461/s1, Figure S1: Results of redundancy analysis (RDA) between strawberry leaf microbial communities and Brix.; Table S1: Read number and final ASV ratio during leaf microbiome preprocessing.
