# Supplementary material for: Changes in the Structure of Strawberry Leaf Surface Bacterial and Fungal Communities by Plant Biostimulants
Source: Microorganisms. 2025 Oct 28;13(11):2461. doi: 10.3390/microorganisms13112461 (PMC12654091; doi:10.3390/microorganisms13112461)
Supplement: Supplementary file 1 [file microorganisms-13-02461-s001.zip › microorganisms-3939300-supplementary.pptx]

## Slide 1
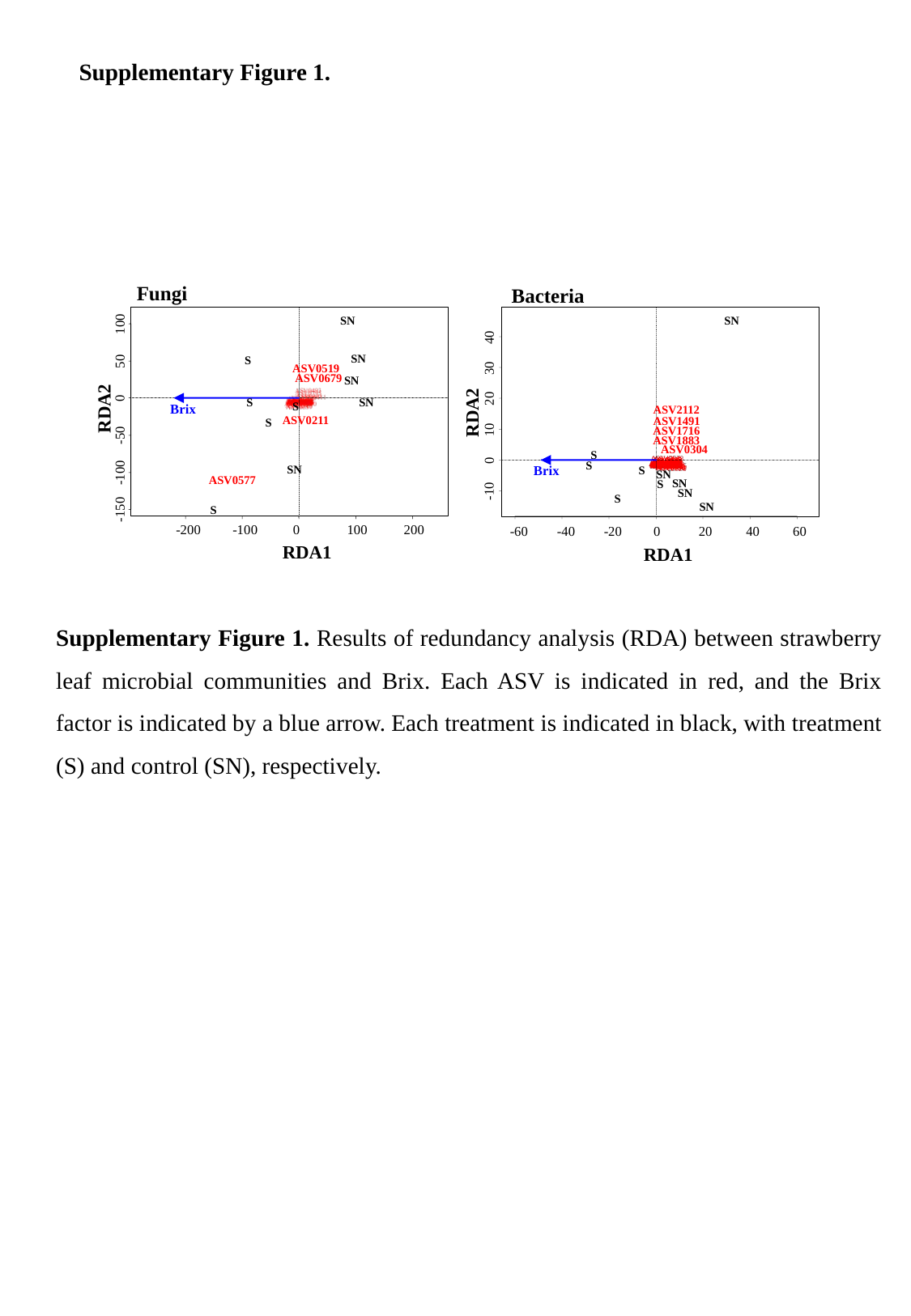

Supplementary Figure 1.
Fungi
Bacteria
SN
40
30
20
RDA2
ASV2112
ASV1491
10
ASV1716
ASV1883
ASV0304
S
0
S
Brix
S
SN
SN
S
-10
SN
S
SN
-60
-40
-20
0
20
40
60
RDA1
SN
100
SN
S
50
ASV0519
ASV0679
SN
0
S
SN
RDA2
S
Brix
ASV0211
S
-50
SN
-100
ASV0577
-150
S
-200
-100
0
100
200
RDA1
Supplementary Figure 1. Results of redundancy analysis (RDA) between strawberry leaf microbial communities and Brix. Each ASV is indicated in red, and the Brix factor is indicated by a blue arrow. Each treatment is indicated in black, with treatment (S) and control (SN), respectively.

## Slide 2
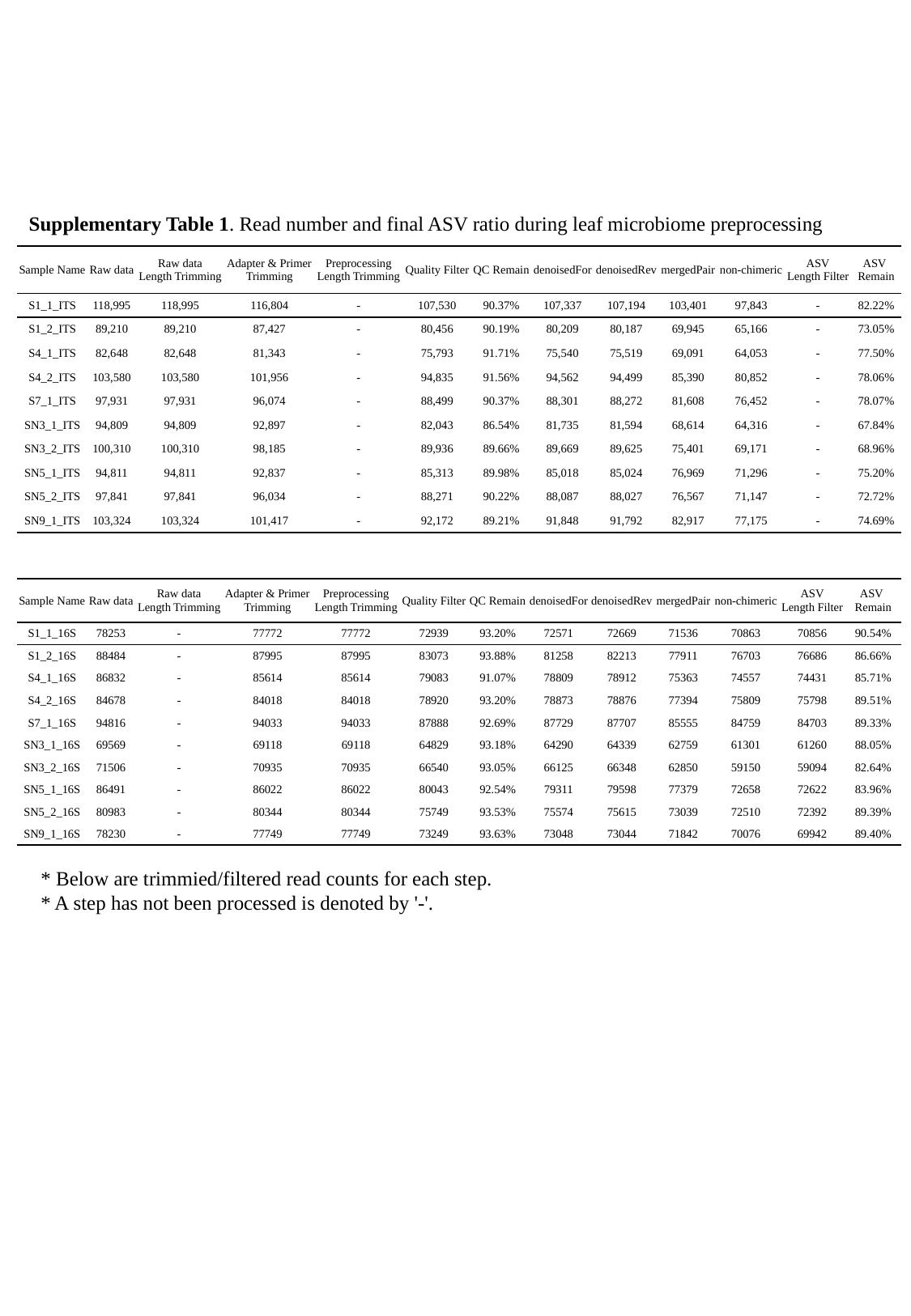

Supplementary Table 1. Read number and final ASV ratio during leaf microbiome preprocessing
| Sample Name | Raw data | Raw data Length Trimming | Adapter & Primer Trimming | Preprocessing Length Trimming | Quality Filter | QC Remain | denoisedFor | denoisedRev | mergedPair | non-chimeric | ASV Length Filter | ASV Remain |
| --- | --- | --- | --- | --- | --- | --- | --- | --- | --- | --- | --- | --- |
| S1\_1\_ITS | 118,995 | 118,995 | 116,804 | - | 107,530 | 90.37% | 107,337 | 107,194 | 103,401 | 97,843 | - | 82.22% |
| S1\_2\_ITS | 89,210 | 89,210 | 87,427 | - | 80,456 | 90.19% | 80,209 | 80,187 | 69,945 | 65,166 | - | 73.05% |
| S4\_1\_ITS | 82,648 | 82,648 | 81,343 | - | 75,793 | 91.71% | 75,540 | 75,519 | 69,091 | 64,053 | - | 77.50% |
| S4\_2\_ITS | 103,580 | 103,580 | 101,956 | - | 94,835 | 91.56% | 94,562 | 94,499 | 85,390 | 80,852 | - | 78.06% |
| S7\_1\_ITS | 97,931 | 97,931 | 96,074 | - | 88,499 | 90.37% | 88,301 | 88,272 | 81,608 | 76,452 | - | 78.07% |
| SN3\_1\_ITS | 94,809 | 94,809 | 92,897 | - | 82,043 | 86.54% | 81,735 | 81,594 | 68,614 | 64,316 | - | 67.84% |
| SN3\_2\_ITS | 100,310 | 100,310 | 98,185 | - | 89,936 | 89.66% | 89,669 | 89,625 | 75,401 | 69,171 | - | 68.96% |
| SN5\_1\_ITS | 94,811 | 94,811 | 92,837 | - | 85,313 | 89.98% | 85,018 | 85,024 | 76,969 | 71,296 | - | 75.20% |
| SN5\_2\_ITS | 97,841 | 97,841 | 96,034 | - | 88,271 | 90.22% | 88,087 | 88,027 | 76,567 | 71,147 | - | 72.72% |
| SN9\_1\_ITS | 103,324 | 103,324 | 101,417 | - | 92,172 | 89.21% | 91,848 | 91,792 | 82,917 | 77,175 | - | 74.69% |
| Sample Name | Raw data | Raw data Length Trimming | Adapter & Primer Trimming | Preprocessing Length Trimming | Quality Filter | QC Remain | denoisedFor | denoisedRev | mergedPair | non-chimeric | ASV Length Filter | ASV Remain |
| --- | --- | --- | --- | --- | --- | --- | --- | --- | --- | --- | --- | --- |
| S1\_1\_16S | 78253 | - | 77772 | 77772 | 72939 | 93.20% | 72571 | 72669 | 71536 | 70863 | 70856 | 90.54% |
| S1\_2\_16S | 88484 | - | 87995 | 87995 | 83073 | 93.88% | 81258 | 82213 | 77911 | 76703 | 76686 | 86.66% |
| S4\_1\_16S | 86832 | - | 85614 | 85614 | 79083 | 91.07% | 78809 | 78912 | 75363 | 74557 | 74431 | 85.71% |
| S4\_2\_16S | 84678 | - | 84018 | 84018 | 78920 | 93.20% | 78873 | 78876 | 77394 | 75809 | 75798 | 89.51% |
| S7\_1\_16S | 94816 | - | 94033 | 94033 | 87888 | 92.69% | 87729 | 87707 | 85555 | 84759 | 84703 | 89.33% |
| SN3\_1\_16S | 69569 | - | 69118 | 69118 | 64829 | 93.18% | 64290 | 64339 | 62759 | 61301 | 61260 | 88.05% |
| SN3\_2\_16S | 71506 | - | 70935 | 70935 | 66540 | 93.05% | 66125 | 66348 | 62850 | 59150 | 59094 | 82.64% |
| SN5\_1\_16S | 86491 | - | 86022 | 86022 | 80043 | 92.54% | 79311 | 79598 | 77379 | 72658 | 72622 | 83.96% |
| SN5\_2\_16S | 80983 | - | 80344 | 80344 | 75749 | 93.53% | 75574 | 75615 | 73039 | 72510 | 72392 | 89.39% |
| SN9\_1\_16S | 78230 | - | 77749 | 77749 | 73249 | 93.63% | 73048 | 73044 | 71842 | 70076 | 69942 | 89.40% |
* Below are trimmied/filtered read counts for each step.
* A step has not been processed is denoted by '-'.
